# Arg-Vasotocin Directly Activates Isotocin Receptors and Induces COX2 Expression in Ovoviviparous Guppies

**DOI:** 10.3389/fendo.2021.617580

**Published:** 2021-04-23

**Authors:** Li Kang Lyu, Jian Shuang Li, Xiao Jie Wang, Yi Jia Yao, Ji Fang Li, Yun Li, Hai Shen Wen, Xin Qi

**Affiliations:** Key Laboratory of Mariculture, Ministry of Education, Ocean University of China, Qingdao, China

**Keywords:** Arg-vasotocin, isotocin receptor, cyclooxygenase, prostaglandin, parturition, guppy

## Abstract

Oxytocin (OT) is a crucial regulator of reproductive behaviors, including parturition in mammals. Arg-vasopressin (AVP) is a nonapeptide homologous to Arg-vasotocin (AVT) in teleosts that has comparable affinity for the OT receptor. In the present study, ovoviviparous guppies (*Poecilia reticulata*) were used to study the effect of AVT on delivery mediated by the activation of prostaglandin (PG) biosynthesis *via* isotocin (IT) receptors (ITRs). One copy each of *it* and *avt* and two copies of *itrs* were identified in guppies. The results of the affinity assay showed that various concentrations of AVT and IT (10^−6^, 10^−7^, and 10^−8^ mol/L) significantly activated *itr1* (P < 0.05). *In vitro* experiments revealed significant upregulation (P < 0.05) of cyclooxygenase 2 (*cox2*), which is the rate-limiting enzyme involved in PG biosynthesis, and *itr1* by AVT and IT. Furthermore, dual *in situ* hybridization detected positive signals for *itr1* and *cox2* at the same site, implying that ITR1 may regulate *cox2* gene expression. Measurement of prostaglandin F_2a_ (PGF_2a_) concentrations showed that AVT induced PGF_2a_ synthesis (P < 0.05) and that the effect of IT was not significant. Finally, intraperitoneal administration of PGF_2a_ significantly induced premature parturition of guppies. This study is the first to identify and characterize AVT and ITRs in guppies. The findings suggest that AVT promotes PG biosynthesis *via* ITR and that PGF_2a_ induces delivery behavior in ovoviviparous guppies.

## Introduction

Oxytocin (OT) is a highly conserved nonapeptide that was the first peptide hormone with determined molecular structure (1). OT has various effects and is involved in many physiological processes, especially reproduction. In mammals, OT has a notable effect on the induction of parturition and smooth muscle contraction and has been used as a medicine in the clinic (2–5). In teleosts, studies of isotocin (IT), a homolog of OT, have been primarily focused on social behaviors, including social decision-making and anxiety behaviors in mosquitofish (*Gambusia affinis*) (6), paternal care and social class in cichlid fish species (7–9) and territory defense, egg care and courtship behaviors in three-spined stickleback (*Gasterosteus aculeatus*) (10). However, the mechanism governing the effect of IT on ovulation in oviparous teleosts has not been determined. IT may influence ovulation in teleosts. OT injection in killifish (*Fundulus heteroclitus*) and seurukan fish (*Osteochilus vittatus*) induces the spawning reflex (11, 12). Injection of a combination of Ovaprim and IT activated ovulation in Hoven’s carp (*Leptobarbus hoevenii*) (13). The functions of IT in the reproduction are limited to the induction of premature parturition in guppy (*Poecilia reticulata*) (14). Other studies suggested that AVT may also regulate sex-related reproductive behaviors.

Arg-vasotocin (AVT) is the homolog of mammalian Arg-vasopressin (AVP). AVT functions in various adaptive behaviors are similar to those of IT. AVT is important for osmoregulation (15), and increasing number of neuroanatomical studies reported that AVT is involved in the regulation of sex-related reproductive behavior (16–18). In teleosts, exogenous administration of AVT to peacock blenny (*Solaria pavo*) induced female courtship behavior and the expression of nuptial coloration (19). Identification of putative functional sites of AVT in the hypothalamic-pituitary-gonad (HPG) axis in chanchita (*Cichlasoma dimerus*) indicated a positive effect on gonadotropin secretion and on the differences in the social status in males (20).

Both OT/IT and AVP/AVT are nonapeptide hormones produced from a common precursor (21) and share seven out of nine amino acids in their primary structure (22). OT and AVP may bind to each other receptors due to strong similarity of their chemical structures (22). A study in Wistar rats demonstrated that peripheral administration of both OT and AVP induced profound effects manifested as a reduction in body temperature and heart rate, and these changes were predominantly mediated by the AVP V1a receptor (V1aR) (23). Peripheral administration of the OT and AVP neuropeptides induced prosocial effects that were prevented by an oxytocin receptor (OTR) antagonist but not a V1aR antagonist, indicating a possible function of AVP *via* OTR (24). With regard to reproduction, both OT and AVP induce uterine contractions in nonpregnant mice (*Mus musculus*) through a mechanism mediated by OTR (25). Overall, these findings indicate possible crosstalk between the OT/IT and AVP/AVT systems.

The OT/OTR system was shown to participate in various physiological processes during the reproductive period (1). OT is important for parturition and acts through its specific receptor (26). OT binding to OTR increases intracellular Ca^2+^ concentration and thus activates smooth muscle contraction (27). Additionally, OT stimulates prostaglandin (PG) synthesis through a more complex pathway linked to the contraction of the myometrium (3, 27). In humans, OT significantly increases prostaglandin E_2_ (PGE_2_) production by increasing the expression of cyclooxygenase 2 (*cox2*) mRNA *via* OTR (28). Treatment of cultured endometrial cells with OT induces COX2 activation and synthesis of PGF_2a_ (29). However, in luteal phase mares, OT downregulated the activities of both COX2 and prostaglandin E_2_ synthase (PTGES) (30). Thus, OT functions involve regulation of PG synthesis, which may be influenced by the reproductive status.

PGs play a central role in the onset of parturition in mammals and are well-known as inflammatory factors and multifunctional fatty acid derivatives comprising a group of C20 metabolites synthesized from arachidonic acid (AA) through a series of enzyme modifications. The synthesis is initiated by the conversion of AA into the intermediate product PGH_2_ by COX1 or COX2 followed by synthesis of five bioactive metabolites, including four PGs (PGE_2_, PGF_2a_, PGD_2_, and PGI_2_) and thromboxane (TX) (31–33). PGF_2a_ induces the contractions and inflammatory responses in the human myometrium (3). Stimulation of OT release from large luteal cells causes additional PGF_2a_ synthesis and release *via* a positive feedback mechanism (34). In teleosts, plasma PGF_2a_ levels in successfully ovulated females are significantly higher than those in unsuccessfully ovulated females in small-scale pacu (*Piaractus mesopotamicus*), indicating the importance of PGF_2a_ in inducing ovulation (35). In goldfish (*Carassius auratus*), PGF_2a_ functions as a blood-borne behavior hormone that induces spawning behavior (36, 37). These experiments suggested that PGF_2a_ has similar activity in inducing ovulation in some externally fertilizing fish.

These studies focused on the effect of IT on teleost spawning behavior mediated by ITR and PGF_2a_. However, unlike other egg-laying teleosts, guppy (*Poecilia reticulata*) uses ovoviviparous reproductive strategy and gives birth to active larval fish directly. A previous study demonstrated that PGF_2a_, IT and AVT significantly shorten the brood interval of guppies (14); however, the molecular mechanism of this effect remains unclear. The present study investigated the molecular mechanism by which AVT and IT stimulate PGF_2a_ synthesis *via* the isotocin receptor (ITR) and subsequently trigger the initiation of parturition in guppies to determine the role of the interaction of AVT and IT with ITR and PGF_2a_ in the regulation in live bearing guppy. The present study is the first to identify and characterize *it*, *avt* and *itrs* in guppies to document the effect of AVT and IT on *cox2* expression and the PGF_2a_ concentration. Finally, the administration of PGF_2a_ to pregnant guppies confirmed the function of AVT-induced PGF_2a_ in parturition in ovoviviparous teleosts.

## Materials and Methods

### Ethics Statement

All procedures involved in the handling and treatment of fish in this study were approved by Animal Research and Ethics Committees of Ocean University of China prior to the initiation of the study. All experiments were performed in accordance with relevant guidelines and regulations.

### RNA Extraction and Reverse Transcription

Total RNA was extracted from the guppy brain and ovaries (n = 3) using TRIzol^®^ reagent (Invitrogen, Carlsbad, USA) for gene cloning. The quantity and purity of the RNA were estimated using a biophotometer (OSTC, Beijing, China) and agarose gel electrophoresis. One microgram of total RNA was reverse transcribed into complementary DNA (cDNA) using a HiScript III RT SuperMix reagent kit (Vazyme, Nanjing, China) according to the manufacturer’s instructions.

### Gene Cloning and Sequence Analysis of Isotocin, Arg-Vasotocin and Isotocin Receptors in Guppies

The open reading frames (ORFs) of guppy *it*, *avt*, and *itrs* were obtained from the genome database (PRJNA238429), and the sequences were confirmed by PCR followed by Sanger sequencing. PCR was performed according to the protocol described in a previous report using cDNA samples from the brain or ovary (38). Briefly, initial denaturation was performed at 94°C for 3 min followed by 35 cycles at 94°C for 30 s, 55°C to 60°C for 30 s, and 72°C for 1 min. The reaction was terminated with an extension for 5 min at 72°C. The products were purified using a TIANgel Midi purification kit (TIANGEN, Beijing, China), subcloned into the pEASY-T1 cloning vector (TransGen Biotech, Beijing, China) and transformed into DH5α cells. Positive clones containing the inserts of the expected size were selected for sequencing to confirm the results. The confirmed sequences were submitted to the NCBI. All primers used in the present study are listed in **Table 1**.

**Table 1:** Sequences of the primers used for dual-fluorescence ISH, plasmid construction and qPCR.

Primers	Sequence (5'-3')
Primers for ORF cloning	
*itr1*-orf-F	ATGGAAACTATTTCCAATG
*itr1*-orf-R	TTACGTGGTGGATGTCTGTGT
*itr2*-orf-F	ATGGAGGAACTTTTACGCGCA
*itr2*-orf-R	TCAGTGCGCGGGCCCCCC
*avt*-orf-F	ATGCATCACTCCCTGCTGTGC
*avt* -orf-R	TCAGTAGTCGTTCTGTCCTCT
*it*-orf-F	TGGCTTTCGGCTTTCTGGGT
*it*-orf-R	AGAGAGACCTTCGGGTAGCG
Primers for preparation of dual-fluorescence ISH probes	
*itr1*-ish-F	CGCAACTTATCTGGGACA
*itr1*-ish-R	CCGTAATACGACTCACTATAGGGAGACATCACGGTGGTTATCTTCG
*cox2*-ish-F	CGCATCCGAGTTCAATAC
*cox2*-ish-R	CCGATTTAGGTGACACTATAGAAGCGTTCAAACGAGGAGTAGGG
Primers for plasmid construction	
*itr1*-orf-PC-F	ACTATAGGGAGACCCAAGCTTATGGAAACTATTTCCAATGAAAGTGA
*itr1*-orf-PC-R	TATAGAATAGGGCCCTCTAGATTACGTGGTGGATGTCTGTGTGA
Primers for qPCR	
*cox2*-F	CGCATCCGAGTTCAATAC
*cox2*-R	TTCAAACGAGGAGTAGGG
*itr1*-F	CGCAACTTATCTGGGACA
*itr1*-R	TCACGGTGGTTATCTTCG
*β-actin*-F	GCCTATCTACGAGGGCTACGC
*β-actin*-R	TTGATGTCACGCACGATTTCC

The signal peptide and precursor cleavage sites of IT and AVT were predicted by SignalP 5.0 (39) and NeuroPred software (40), respectively. Seven putative transmembrane domains were predicted by the TMHMM server v. 2.0 (http://www.cbs.dtu.dk/services/TMHMM-2.0/). Multiple sequences were aligned and analyzed using Clustal X software (41), and phylogenetic trees were constructed using MEGA 6 software (42).

### Colocalization of Guppy *itr1* and *cox2* Using Dual-Fluorescence *In Situ* Hybridization (ISH)

Dual-fluorescence *in situ* hybridization (ISH) for *itr1* and *cox2* was performed to confirm the regulatory effect of AVT on *cox2 via* ITR1 using previously described methods with modifications (43, 44). Briefly, 10 guppy ovaries at various development stages were collected, fixed with buffered 4% paraformaldehyde for 24 h and subsequently embedded in paraffin. Next, 7-mm thick sections were cut for ISH. For double ISH, the probes for *itr1* and *cox2* were labeled with digoxigenin (DIG) and biotin (Roche Diagnostics, Mannheim, Germany), respectively. After hybridization with a mixture of both probes and post-hybridization steps, the sections were washed and blocked with blocking buffer (10% goat serum, Invitrogen, Carlsbad, USA). The sections were incubated with a horseradish peroxidase (HRP)-conjugated anti-DIG secondary antibody (diluted 1:500 with blocking buffer, Roche Diagnostics, Mannheim, Germany) and rinsed twice with sterile phosphate-buffered saline (PBS) for 5 min each time; then, chromogenic reactions were performed using a tyramide kit with Alexa Fluor 488 (Invitrogen) for 30 min. The second fluorescence detection started only when the first reaction appeared to produce appropriate results. The sections were incubated with 3% hydrogen peroxide for 1 h to inactivate HRP conjugated to anti-DIG antibody. After several rinsing steps, the sections were incubated with HRP-conjugated streptavidin (Proteintech, Chicago, USA). The final chromogenic reaction was performed using a tyramide kit with Alexa Fluor 594 (Invitrogen) for 30 min and stopped by adding working solution of stop reagent (Invitrogen, Carlsbad, USA) to detect the signal. The sections were mounted in antifade mounting medium (Beyotime, Shanghai, China) after nuclear staining with DAPI for 10 s (10 μg/mL, Solarbio, Beijing, China). Images were captured using an Olympus BX53F fluorescence microscope (Olympus, Japan).

### *In Vitro* Ovary Incubation Assays

The guppy nonapeptides AVT and IT were synthesized by GL Biochem (Shanghai, China). The purity of the synthesized peptides was determined to be >95% using analytical HPLC. The nonapeptides were dissolved in dimethyl sulfoxide (DMSO, Solarbio, Beijing, China). Tissue culture was performed mainly using an established protocol (45). Briefly, whole ovaries from five fertilized female guppies (1.15 ± 0.09 g) per group were collected and washed three times with PBS containing 100 U/mL penicillin, 1 mg/mL streptomycin and 1.25 U/mL nystatin. After preincubation with Leibovitz’s L-15 medium in a 12-well plate at 28°C for 2 h, the ovarian fragments were incubated in fresh medium containing various doses of AVT or IT (10^−5^, 10^−6^, or 10^−7^ mol/L) for 3 h or with 10^−5^ mol/L AVT or IT for various times (3, 6, or 9 h). The fragments incubated with DMSO were used as the controls. At the end of the incubation, the fragments were collected and stored at −80°C for RNA extraction and qPCR analysis.

### Quantitative Real-Time PCR

The expression of *itr1* and *cox2* in guppies was analyzed by quantitative real-time PCR (qPCR) with specific primers. The samples were generated from ovary fragments incubated with various concentrations of the stimulatory agents at various time points as indicated. All cDNA products obtained by RNA extraction and reverse transcription were diluted to 500 ng/μl. The 20-μL qPCR reaction mixture contained 2 μL of cDNA templates, 0.4 μL of both primers, 10 μL of KAPA SYBR^®^FAST qPCR Master Mix (2X), 0.4 μL of 6-carboxy-X-rhodamine (ROX) and 6.8 μL of RNase-free water. PCR amplification was performed in a 96-well optical plate at 95°C for 30 s followed by 40 cycles at 95°C for 5 s and 58°C for 30 s, and a final extension was performed at 72°C for 2 min. qPCR was performed using a StepOne Plus real-time PCR system (Applied Biosystems), and the 2^−ΔΔCT^ method was used to calculate the gene expression levels.

### Plasmid Construction and Luciferase Assay

The human embryonic kidney 293T (HEK-293T) cell line was used for the binding activity assays. The ORF of the guppy *itr1* cDNA was subcloned into the pcDNA3.1a expression vector (Invitrogen, Carlsbad, USA). Prior to the transfection, 293T cells were maintained at 37°C in DMEM containing 10% fetal bovine serum (FBS) (Thermo Fisher Scientific, Massachusetts, USA). Twenty hours before the transfection, 1 × 10^5^ cells were seeded into the wells of 24-well tissue culture plates. Then, 500 ng of pCRE-Luc reporter plasmid, 300 ng of pcDNA3.1-itr1 and 50 ng of pRL-CMV (to normalize transfection efficiency) containing the Renilla luciferase sequence were transiently cotransfected into the cells in 500 μL of serum-free medium using Lipofectamine reagent (Invitrogen, Carlsbad, USA). Six hours after the transfection, the cells were incubated in DMEM (10% FBS) for 12 h and subsequently treated with the vehicle or various concentrations of IT and AVT (10^−6^, 10^−7^, or 10^−8^ mol/L) for an additional 6 h. Each treatment was replicated in four wells. Then, the cells were harvested, and luciferase activity assays were performed using a dual-luciferase kit (Promega, Wisconsin, USA).

### ELISA Measurement of the PGF_2a_ Concentration

To assess the PGF_2a_ concentrations after IT and AVT injections, 24 pregnant guppies were intraperitoneally injected with IT (1 μg/g), AVT (1 μg/g) or the same volume of saline solution. Tissue homogenates from each individual were collected 3 h after the injection. The homogenate was obtained by centrifugation of the samples at 12,000 g for 10 min after an overnight incubation at 4°C, and the PGF_2a_ level was measured in duplicate samples using commercial ELISA kits (Runyu, Shanghai, China) according to the manufacturer’s instructions.

### PGF_2a_ Injection and Parturition Behavioral Assay

Parturition behavioral assays were conducted in 2-L rectangular tanks. Guppies were selected and treated 20 days after the last delivery, and 10 individuals were housed in two 5-L water tanks with water temperature of 28°C before the injection. One pregnant individual from each tank was acclimated for 2 days to standardize the effect of environmental changes. PGF_2a_ (Shanghai Yuanye Bio-Technology, China) was injected at a concentration of 1,000 ng/g body wet weight to verify whether PGF_2a_ has a direct effect on parturition. PGF_2a_ was dissolved in ethanol at a concentration of 100 μg/mL, as described in a previous study of PG administration in guppy (14), with several modifications. Individuals in the control group were injected with an equal volume of ethanol. All reactions were recorded with a digital video camera. The treatment and control were administered at the same time. The experiment was replicated three times. The offspring were collected and imaged using a microscope (Optec, China) to analyze the morphology.

### Statistical Analysis

All data are presented as the mean values ± S.E.M. The PGF_2a_ concentration and gene expression in response to various concentrations of the hormones and at various treatment times were analyzed using one-way ANOVA followed by Duncan’s and Dunnett’s T3 multiple range tests. Gene expression in guppy treated with various hormones at the same time points were analyzed using independent sample T test. P-values <0.05 were considered to be significant. All statistical analyses were performed using SPSS 19.0 software (SPSS, Chicago, IL, USA).

## Results

### Gene Cloning and Sequence Analysis of AVT, IT, and ITRs in Guppies

**Figure 1:**
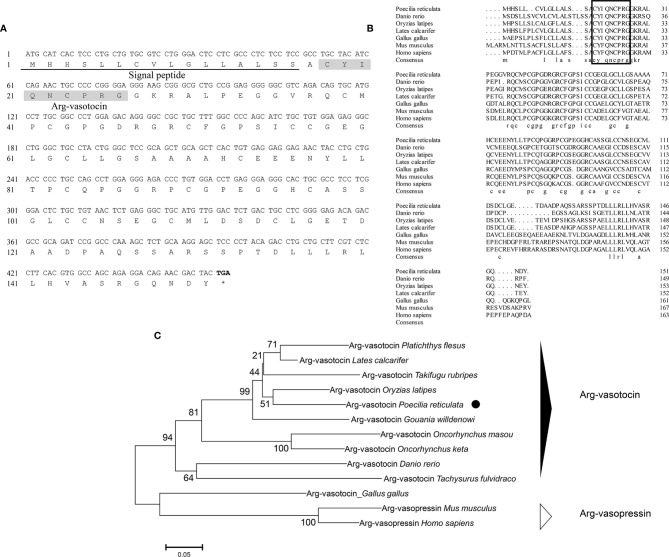
**(A)** Nucleotide and deduced amino acid sequences of guppy AVT. The putative signal peptide is underlined. The putative AVT peptide is shaded. **(B)** Multiple comparisons of amino acid sequences of AVP/AVT precursors. The boxed letters indicate the sequence of mature AVP/AVT. **(C)** Phylogenetic analysis of AVP/AVT precursors in different species. Phylogenetic tree was constructed using MEGA 6 software and the neighbor-joining method. The data were resampled with 1,000 bootstrap replicates. The accession numbers of each sequence are as follows: *Oryzias latipes* (BAM15897.1), *Lates calcatifer* (XM_018674883.1), *Takifugu rubripes* (AAC60293.1), *Danio renio* (NP_840078.1), *Homo sapiens* (AAA61291.1), *Gallus gallus* (NP_990516.1), *Mus musculus* (AAC42027.1), *Tachysurus fulvidraco* (XM_027167236), *Oncorhynchus masou* (AAC60743.1), *Oncorhynchus keta* (CAA35205.1), *Platichthys flesus* (BAA98140.1), and *Gouania willdenowi* (XM_02457450.1).

Genomic data mining and gene cloning showed that the open reading frame (ORF) of *avt* (MW050982) is 456 bp and encodes a 151-amino acid (aa) precursor with a predicted signal peptide of 16 aa (**Figure 1A** **)**. The precursor cleavage site analysis showed that the putative nonapeptide is located from aa 18 to aa 27 **(** **Figure 1A** **)**. Comparison of the deduced amino acid sequences revealed that teleost AVT nonapeptides are highly conserved with avian AVTs and human AVP, except for a mutation site at position 3 (Gln/Ser) **(** **Figure 1B** **)**. A phylogenetic tree of AVP precursors was constructed, and AVTs from teleosts and avians were clustered into a single clade, along with mammalian AVPs **(** **Figure 1C** **)**.

**Figure 2:**
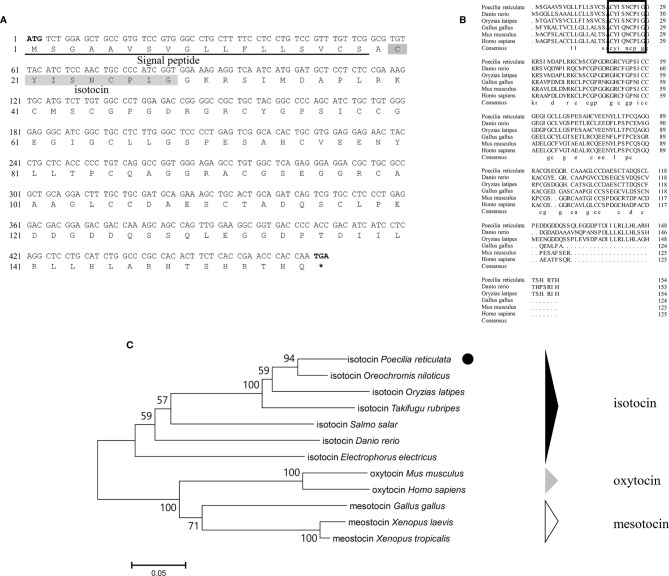
**(A)** Nucleotide and deduced amino acid sequences of guppy IT. The putative signal peptide is underlined. The putative IT peptide is shaded. **(B)** Multiple comparisons of the amino acid sequences of OT/IT/MT precursors. The boxed letters indicate the sequence of mature OT/IT/MT. **(C)** Phylogenetic analysis of IT precursors in different species. The phylogenetic tree was constructed using MEGA 6 software and the neighbor-joining method. The data were resampled with 1,000 bootstrap replicates. The accession numbers of each sequence are as follows: *Oryzias latipes* (NP_001265759.1), *Salmo salar* (ABU80634.1), *Takifugu rubripes* (AAC60289.1), *Danio renio* (AAL50209.1), *Homo sapiens* (NP_000906.1), *Gallus gallus* (QCX08109.1), *Mus musculus* (NP_035155.1), *Electrophorus electricus* (XP_026855497.1), *Oreochromis niloticus* (XP_003446141.1), *Xenopus laevis* (XP_018080922.1), and *Xenopus tropicalis* (XP_002936405.1).

**Figure 3:**
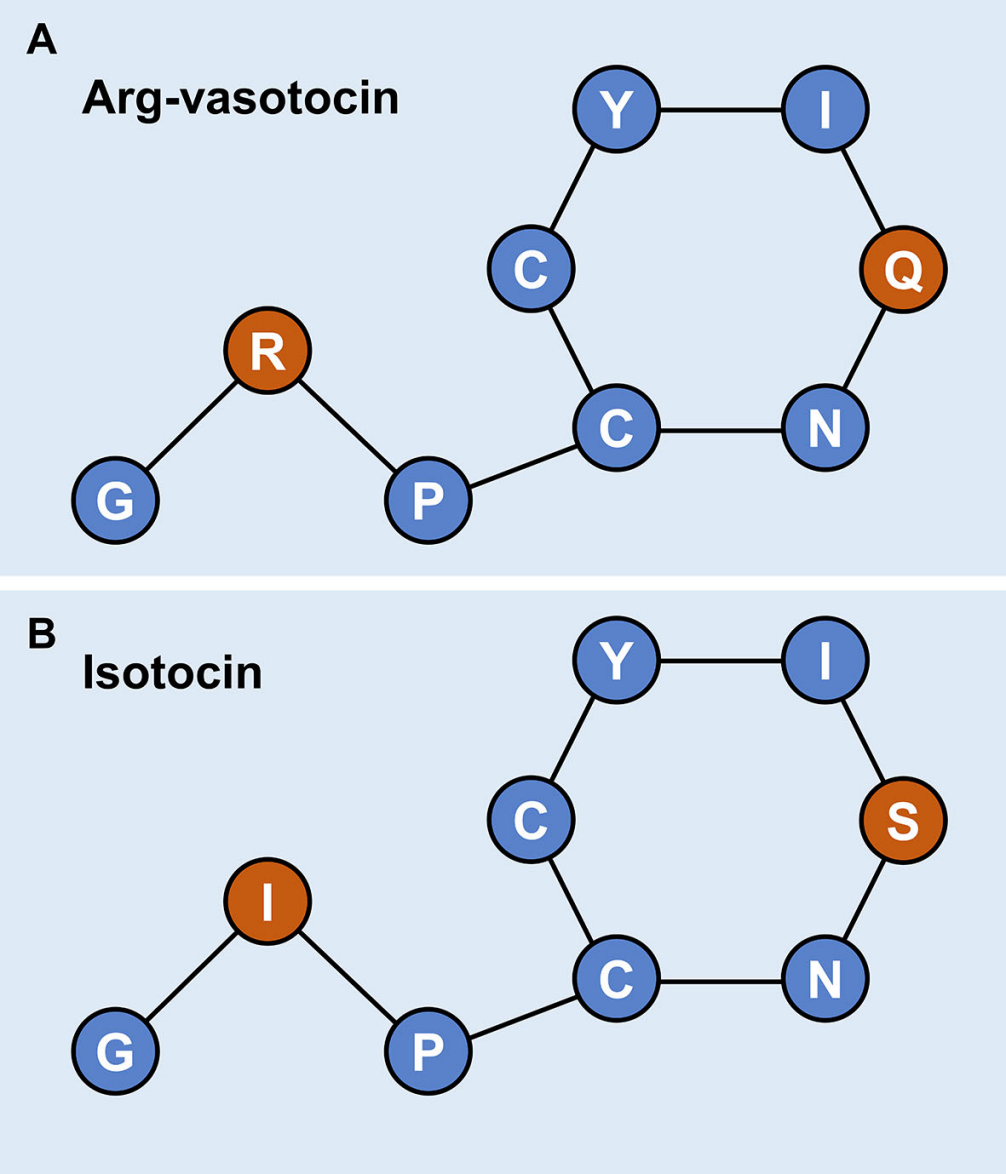
Schematic diagrams of the primary structures of guppy AVT **(A)** and IT **(B)**. Different amino acids at positions 4 and 8 are highlighted in red.

Genomic data mining and gene cloning indicated that the ORF (MW050983) of *it* is 468 bp and encodes a 155 aa precursor with a predicted signal peptide of 18 aa (**Figure 2A** **)**. The putative nonapeptide extends from 20 aa to 28 aa based on precursor cleavage site analysis **(** **Figure 2A** **)**. Comparison of the deduced amino acid sequences indicated that the IT nonapeptides are highly conserved among teleosts and similar to human OT and chicken mesotocin (MT), except for two mutation sites at positions 4 (Gln/Ser) and 8 (Leu/Ile) **(** **Figure 2B** **)**. A phylogenetic tree of OT-like precursors revealed the clustering of teleost ITs into a single clade with mammalian OTs and avian and tetrapod MTs **(** **Figure 2C** **)**. As neuropeptides with nine amino acids, both AVT and IT show high sequence conservation, except for Gln/Ser at position 4 and Arg/Ile at position 8. Both peptides contain disulfide bonds between cysteine (Cys) residues at positions 1 and 6 (**Figure 3**).

**Figure 4:**
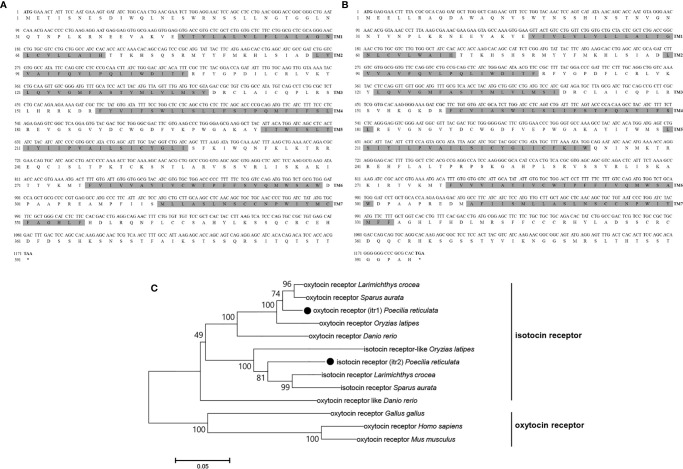
Nucleotide and deduced amino acid sequences of guppy ITR1 **(A)** and ITR2 **(B)**. The shading represents transmembrane domains. **(C)** Phylogenetic analysis of the OTR family in different species. Phylogenetic tree was constructed using MEGA 6 software and the neighbor-joining method. The data were resampled with 1,000 bootstrap replicates. The accession numbers of each sequence are as follows: *Carassius auratus* (XP_026116839.1), *Salvelinus alpinus* (XP_023846756.1), *Mastacembelus armatus* (XP_026163763.1), ITR1 *Oncorhynchus mykiss* (XP_021466000.1), ITR2 *Oncorhynchus mykiss* (XP_021465666.1), ITR1 *Seriola lalandi dorsalis* (XP_023257499.1), ITR2 *Seriola lalandi dorsalis* (XP_023265951.1), *Cynoglossus semilaevis* (XP_016892293.1), ITR1 *Danio rerio* (NP_001186298.1), ITR2 *Danio rerio* (NP_001186299.1), *Gallus gallus* (NP_001026740.1), *Homo sapiens* (NP_000907.2), *Mus musculus* (NP_001074616.1), *Oryzias latipes* (NP_001243561.1), *Paramormyrops kingsleyae* (XP_023661236.1), *Perca flavescens* (XP_028431436.1), *Stegastes partitus* (NP_001281113.1), *Poecilia formosa* (XP_007570475.1), and *Paralichthys olivaceus* (XP_019942082.1).

Genomic data revealed 2 *oxytocin-like receptor* genes in guppies. The size of the *oxytocin receptor* gene is 1,173 bp, and the gene encodes a 390-aa G protein-coupled receptor; the classical 7-transmembrane domain structure is annotated in **Figure 4A**. The ORF of *itr* is 1,188 bp, coding for a 395-aa G protein-coupled receptor with seven transmembrane domains (**Figure 4B**).

Teleosts clustered together in the phylogenetic tree of both OT-like receptors. Teleosts, including zebrafish, guppy, medaka (*Oryzias latipes*), large yellow croaker (*Larimichthys crocea*), and gilt-head bream (*Sparus aurata*), express two types of the receptors, which were previously known as OTRs and ITRs. In the present study on guppies, the oxytocin receptor was renamed isotocin receptor 1 (ITR1, MN725111), and the isotocin receptor was renamed isotocin receptor 2 (ITR2, MW050984). However, oxytocin-like receptors in non-teleosts display greater evolutionary distance, suggesting that they are relatively conserved in teleosts **(** **Figure 4C** **)**.

### cAMP Response Element-Luciferase-Based Characterization of the Effects of AVT on Guppy ITR

**Figure 5:**
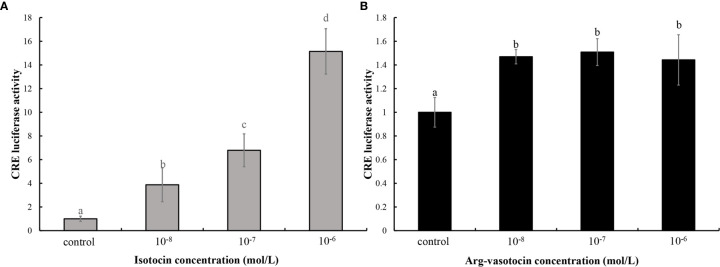
The assay of IT **(A)** and AVT **(B)** binding to ITR1. The relative CRE luciferase activity in the presence of various concentrations of IT (10^−6^, 10^−7^, or 10^−8^ mol/L) and AVT (10^−6^, 10^−7^, or 10^−8^ mol/L) was determined by measuring firefly and Renilla luciferase activities normalized to the values in the control group. Bars represent the mean values ± SEM, and different letters indicate significant differences (P < 0.05).

A binding assay was performed using the cAMP response element (CRE)-luciferase reporter assay system to determine whether guppy IT and AVT bind ITR1 and regulate the downstream gene expression. The ORF of *itr1* (pcDNA3.1a), pGL3 (CRE-luciferase), and pRL-TK were cotransfected into HEK293T cells, which were incubated with various concentrations of IT and AVT for 24 h. Both IT and AVT at the concentrations of 10^−6^, 10^−7^, and 10^−8^ mol/L significantly activated ITR1 (P <0.05) (**Figures 5A, B** **)**. The CRE-luciferase reporter assays demonstrated a dose-dependent response to the IT injection, and the luciferase activity corresponding to ITR1 induction by AVT was not dose-dependent.

### Effects of IT and AVT on the Levels of *cox2* and *itr1* mRNAs in the Guppy Ovaries

**Figure 6:**
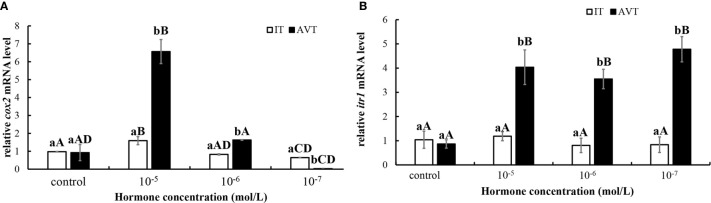
Dose-dependent effects of AVT and IT on the expression of *cox2* and *itr1* mRNAs in guppy ovary fragments. Ovary fragments were incubated with AVT or IT at the concentrations of 10^−5^ mol/L, 10^−6^ mol/L or 10^−7^ mol/L for 3 h, and the levels of *cox2* **(A)** and *itr1* **(B)** mRNAs were measured using qPCR (n=5). The results are presented as the mean ± SEM and as fold changes in the expression levels versus the corresponding controls. Different capital letters indicate significant differences between the groups treated with each concentration of a hormone (P < 0.05). Different lowercase letters indicate significant differences between the two hormone treatments at the same concentration (P < 0.05).

**Figure 7:**
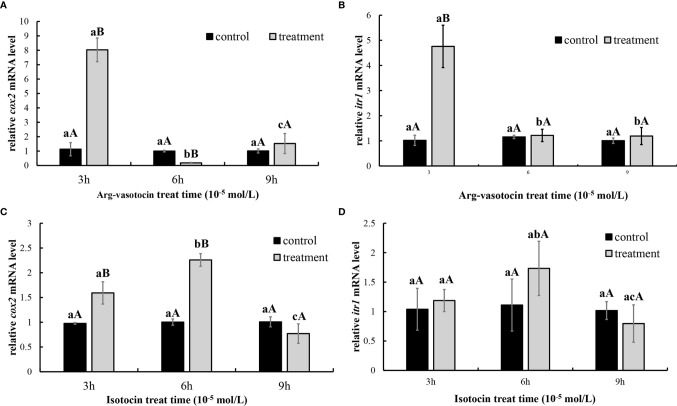
Time-dependent effects of AVT and IT on the expression of *cox2* and *itr1* mRNAs in guppy ovary fragments. The levels of the *cox2* and *itr1* mRNAs were measured using qPCR (n=5). **(A, B)** Ovary fragments were incubated with AVT (10^−5^ mol/L) for 3, 6, or 9 h. **(C, D)** Ovary fragments were incubated with IT (10^−5^ mol/L) for 3, 6, or 9 h. The results are presented as the mean ± SEM and as fold changes in the expression levels versus the corresponding controls. Different capital letters indicate significant differences at the same time points between the treatment and control groups (P < 0.05). Different lowercase letters indicate significant differences in the corresponding control or treatment groups (P < 0.05).

*In vitro* incubation of ovarian fragments was performed to evaluate the effects of IT and AVT on the expression of *cox2* and *itr1* mRNAs. The sequences of *cox2* (XM_008415162.2) and *β-actin* (EU143772.1) were identified using the NCBI database. qPCR was performed to measure the expression of *cox2* and *itr1*. *β-Actin* was used as an internal control. The expression of *cox2* was significantly upregulated (P < 0.05) in the presence of high concentrations of AVT (10^−5^ mol/L) compared with that in the control group and in the samples treated with IT (**Figure 6A**). The expression of *itr1* was also significantly increased (P<0.05) by all concentrations of AVT compared with that in the samples treated with IT **(** **Figure 6B**). The results of time-dependent treatment experiments indicated that the expression levels of *cox2* and *itr1* were significantly induced (P<0.05) at 3 h and rapidly decreased (P < 0.05) at 6 h before returning to the normal levels at 9 h in the presence of a high concentration of AVT (10^−5^ mol/L) (**Figures 7A, B** **)**. In contrast to AVT stimulation, the expression levels of *cox2* and *itr1* were significantly induced (P < 0.05) at 6 h and rapidly decreased (P < 0.05) at 9 h, returning to the normal levels at 9 h in the presence of a high concentration of IT (10^−5^ mol/L) (**Figures 7C, D** **)**.

### Colocalization of *itr1* and *cox2* in the Ovaries of Female Guppies

**Figure 8:**
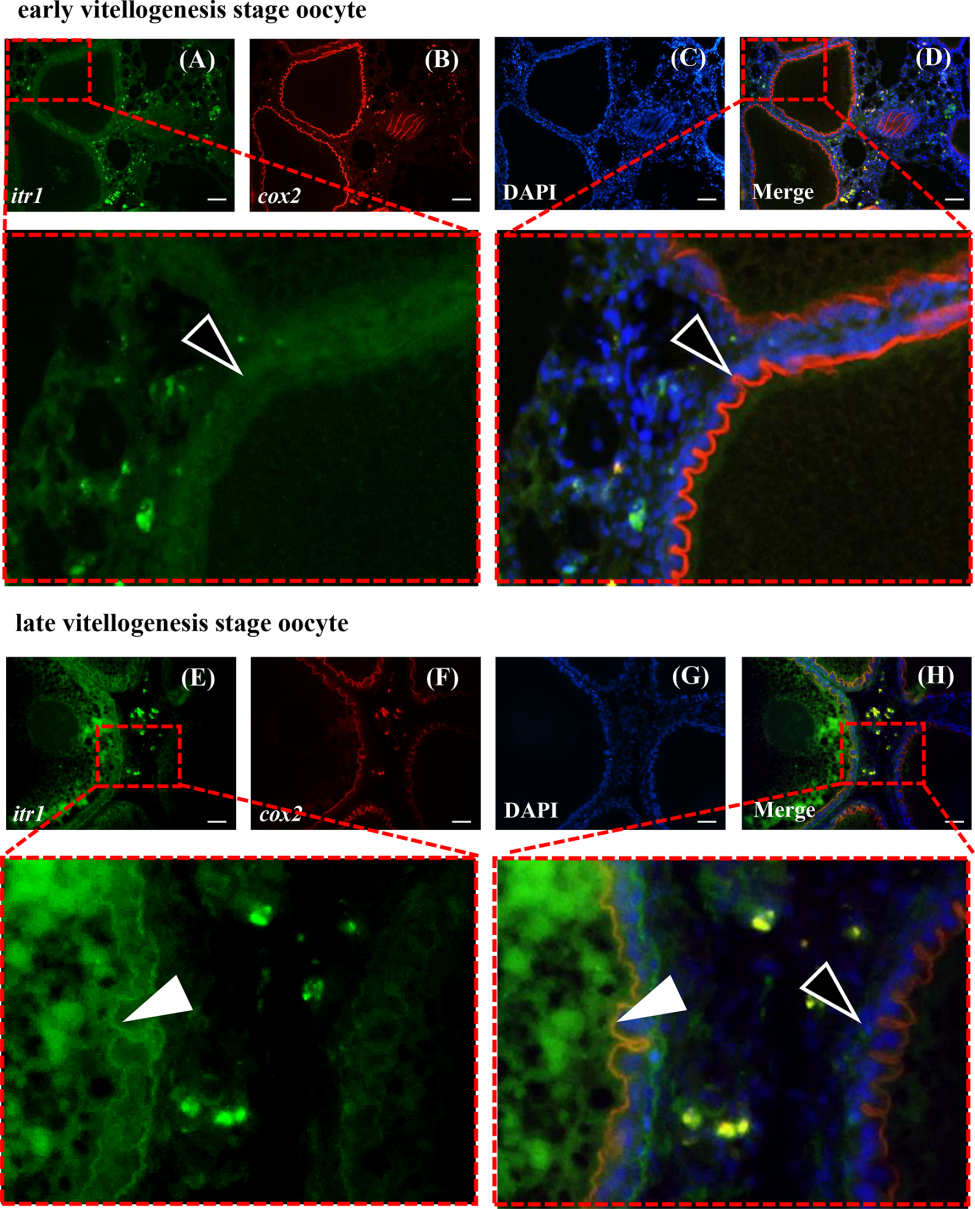
Dual-fluorescence ISH for *itr1* (green, stained with Alexa Fluor 488) and *cox2* (red, stained with Alexa Fluor 594) in guppy ovaries at different developmental stages. **(A–D)** ISH staining in the early vitellogenesis stage. **(E–H)** ISH staining in the late vitellogenesis stage. The *itr1* signal overlapping with the *cox2* signal in the late vitellogenesis stage in the follicular cell layer is indicated by white arrowheads. The single cox2 signal in the early vitellogenesis stage in the follicular layer is indicated by open arrowheads. Nuclei were stained with DAPI (blue). Scale bar, 20 μm.

Dual-fluorescence ISH of *itr1* and *cox2* was performed in the ovary at two different developmental stages to determine possible direct regulatory effect of AVT on *cox2* expression mediated by ITR1. As shown in **Figure 8**, *itr1*-and *cox2*-positive signals were present in the inner follicular cell layer of late vitellogenesis-stage oocytes, indicating that the individual was fertilizable and ready for parturition and that ITR1 may regulate the expression of *cox2* (**Figure 8H**). However, positive signal of *itr1* was not detected in early vitellogenesis-stage oocytes (**Figure 8D**), suggesting that *itr1* expression was significantly influenced by oocyte development stage. On the other hand, positive signals of *itr1* were observed in ovarian stromal cells. Follicles at both stages are indicated with white arrowheads (late vitellogenesis stage oocyte follicles) and open arrowheads (early vitellogenesis stage oocyte follicles) in **Figure 8H**, showing that *itr1* is only expressed in the follicular layer of late vitellogenesis-stage oocytes.

### Analysis of PGF_2a_ Concentrations in IT-and AVT-Treated Guppies

**Figure 9:**
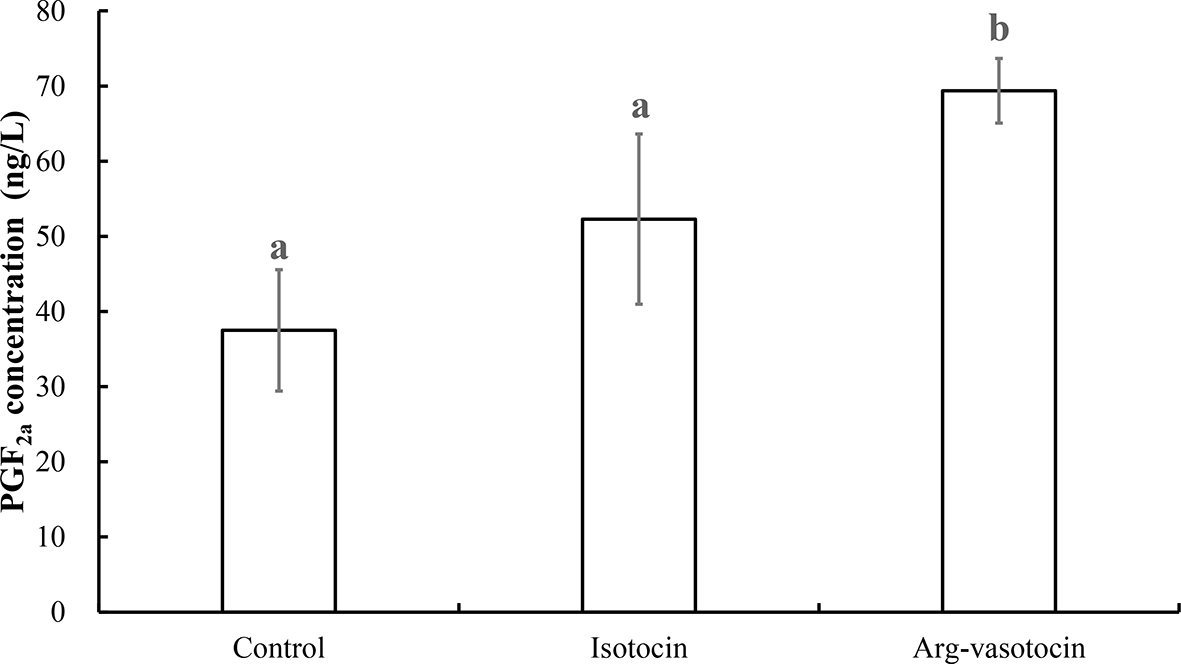
Measurement of PGF_2a_ concentrations after IP injections of IT (1 μg/g), AVT (1 μg/g) or the same volume of saline (control). Eight individuals were analyzed in each group. Different lowercase letters indicate significant differences between the treatment groups (P < 0.05).

PGF_2a_ concentrations were measured to test the effect of neuropeptides on PGF_2a_ biosynthesis in guppy. AVT induced PGF_2a_ synthesis (P < 0.05) compared with that in the control group and IT group 3 h after intraperitoneal (IP) injection. The values in the IT and control groups were not significantly different from each other (**Figure 9**).

### Administration of PGF_2a_ to Pregnant Guppies Induced Premature Parturition

An IP injection of PGF_2a_ (1,000 ng/g body wet weight) into pregnant guppies induced parturition behavior in immature larval fish. **Supplementary Video 1** shows that the first delivery occurred approximately 1 h after the IP injection (9:43 AM local time), and no clear variations in parturition behavior were observed in the control, IT-injected (500 ng/g) and AVT-injected groups (500 ng/g) (data not shown). Up to three dead larval fish at most were observed after PGF_2a_ injection in a guppy. Eleven dead larval fish were observed.

**Figure 10:**
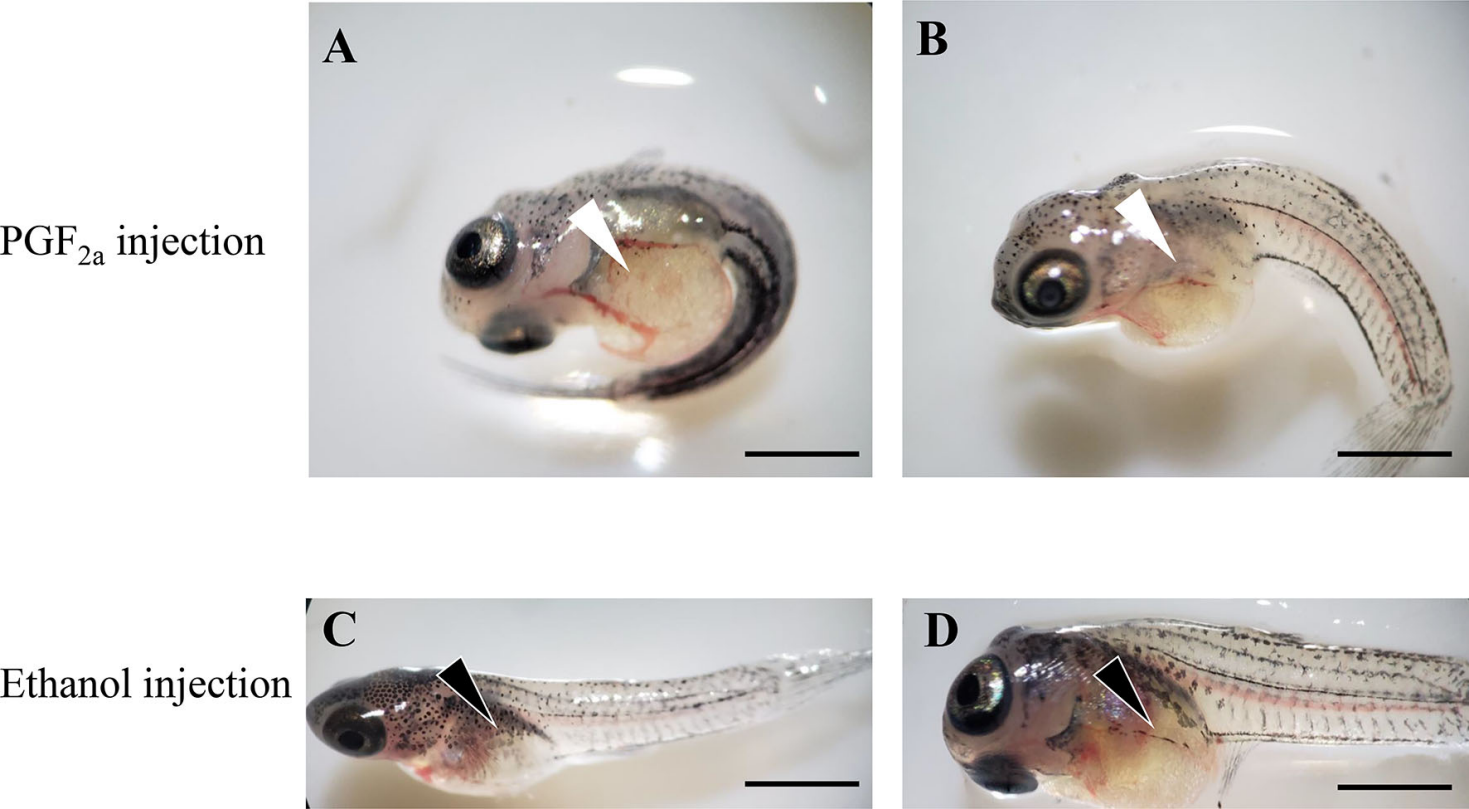
Offspring of pregnant guppies injected with PGF_2a_ (1,000 ng/g) or ethanol (vehicle control). The PGF_2a_ injection induced premature parturition, and larval fish presented larger yolk sac (white arrowheads) and curly bodies **(A, B)**. n=5. Normal larval fish from ethanol-injected pregnant guppies presented a regular yolk sac (black arrowheads) and stretched bodies **(C, D)**. n=5. Scale bar, 1 mm.

PGF_2a_ administration significantly induced the premature parturition of guppies. As shown in **Figure 10**, premature larval fishes retained a larger yolk sac and lower melanin distribution on the body compared with those in the control group (ethanol). The bodies of the larval fish in the control group showed considerably more stretching than those in the PGF_2a_-injected group due to the lack of skeletal muscle development under premature conditions.

## Discussion

Nonapeptide hormones have been identified in the endocrine system in various vertebrates. AVP and its homolog AVT are highly conserved, except position 3, which contains Phe in AVP and Ile in AVT. These peptides are named based on the presence of Arg at position 8. Comparison of OT and IT indicated that OT contains Gln at position 4 and Leu at position 8, and these amino acids are replaced with Ser and Ile, respectively, in IT. In teleosts, which are the predecessors of land vertebrates, amino acid sequences of the AVT and IT nonapeptides share homology, except positions 4 and 8 (1). Thus, two evolutionary lineages have been proposed, the IT-OT line and AVT-AVP line, and both lineages are associated with reproductive functions and behaviors (1, 22). High conservation between IT and AVT was confirmed in the present study by gene cloning and sequence analysis of an IT precursor, an AVT precursor and two ITRs of guppy. The predicted conserved disulfide bridge was formed by Cys residues 1 and 6. Nonapeptides were classified into AVT- and OT-like families according to the 8th amino acid, which is essential for activating the corresponding receptors (1, 46). Basic amino acids, such as Arg, are present at this position in AVT, and neutral amino acids are present in OT-like peptides (1, 46).

In teleosts, the production of IT is primarily observed in the preoptic area (POA); however, ITRs are distributed in various tissues (47). The present study in guppies identified and cloned two *itrs* in agreement with reports in other teleosts, including zebrafish (48), medaka (49) and bicolor damselfish (*Stegastes partitus*) *(* 50). These patterns originated due to two rounds of whole-genome duplication events in vertebrates (2R), which produced one *otr* and five *avp* receptors, and the third round in teleosts (3R) subsequently produced two local duplicates of *itrs* (51). In mammals, one OT receptor (OTR) and three AVP receptors (V1aR, V1bR, and V2R) have been identified in various peripheral tissues (52, 53). Regardless of the similarity of these receptors (25%), the selectivity of OT and AVP for these receptors varies between species and tissues (22, 54). Notably, AVP has similar affinity for OTR, V1aR, and V1bR (22, 55, 56). The OT receptor has the same affinity to hormones with cyclic structures and Arg-8 (AVT) or Leu-8 (OT) (1).

In vertebrates, OT has been shown to stimulate PG synthesis by upregulating *cox2* gene expression to induce the conversion of AA to a PG precursor (3, 27, 28). Further studies showed that in cultured endometrial explants, *cox2* mRNA expression and PGF_2a_ synthesis were increased by OT stimulation and were abolished by the OT antagonist atosiban (29). Similarly, in pre-labor human amnion epithelial cells, incubation with OT resulted in a significant increase in PGE_2_ levels mediated by upregulation of *cox2* expression (28). In addition, the synthesis of PGs was observed in the follicles of human ovaries (57). These studies demonstrated that OT upregulates the *cox2* gene by signaling *via* OTR. Similarly, the studies in teleosts were focused on the role of PGs in the reproductive process, including follicle rupture, ovulation, and spawning (58–60). Notably, a study on catfish (*Heteropneustes fossilis*) demonstrated that AVT is the main factor stimulating PGF_2a_ synthesis (61). However, it is not known whether AVT stimulates PGF_2a_ secretion *via* OTR similar to OT/IT. The results of the present study indicated that *itr1* and *cox2* are colocalized in the inner follicular cell layer that produces PGs. Furthermore, merged *itr1*/*cox2* signals were also observed in ovarian stromal cells, where PGs were reported to play a role in the immune reaction (31).

The CRE-luciferase assay was performed to evaluate the affinity of AVT and IT for ITR1 and to confirm our hypothesis that AVT regulates *cox2* mRNA expression *via* ITR1. The results showed that both IT and AVT bind to ITR1; however, AVT does not display dose-dependent effects. These data are supported by previous studies showing that AVT activates teleost ITRs with lower potency than that of IT (62). AVT has a similar affinity for OTR, V1aR and V1bR (22). Then, we stimulated cultured ovary with AVT and IT *in vitro*. As expected, both *cox2* and *itr1* were upregulated by AVT and IT treatments; however, AVT treatment resulted in faster and stronger transcriptional regulation of *cox2* and *itr1* than that of IT. Measurement of PGF_2a_ concentrations showed that AVT was more efficient inducer of PG synthesis. In humans, OT regulates the expression of its own mRNA through the transcription factor nuclear factor kappa B (NF-κB) and PGs in a positive feedback mechanism. A previous clinical analysis showed that the expression of *cox2* and *otr* was high in women with high NF-κB activity (63). Similar results were obtained in primary human amnion epithelial cells incubated with IL-1β, which is an inflammatory factor that activates NF-κB, resulting in rapid upregulation of *otr *(28). Similarly, our results suggested a conserved parturition mechanism in mammals and live bearing teleost guppies. Notably, OT had opposite effects on luteal phase mares; however, the levels of a series of enzymes involved in PG synthesis, including *cox2*, were decreased after OT administration (30). The data of dual ISH indicated that *itr* localization followed a stage-related expression pattern. This result may explain why OT/IT has different functions in ovaries at different developmental stages.

*In vivo* injection of PGF_2a_ was performed to confirm its function in inducing preterm parturition. Similar to a previous study (14), prematurely delivered fry had a shorter body length and larger yolk sac. Interestingly, the results of this study indicated that a high concentration (500 ng/g), but not a low concentration (50 ng/g), of AVT can induce premature birth in guppy. However, in the present study, injection of AVT (500 ng/g) did not induce premature birth, and direct injection of PGF_2a_ was able to significantly induce premature birth. The differences between individuals and gestation period may be responsible for variability of the results. A study on teleost ovulation reported that PGF_2a_ was more effective than AVT in inducing ovulation in a dose- and duration-dependent manner (64). Considering that stimulation of guppy with AVT increased PGF_2a_ concentration and did not activate premature birth, PGF_2a_ may be more effective than AVT in induction of delivery because AVT is located upstream in the positive feedback regulation of PG synthesis (luteinizing hormone (human chorionic gonadotropin) > 17,20β-dixydroxy-4-pregnen-3-on (DP) < > AVT > PGs > final oocyte maturation (FOM)/ovulation) (64). The results also revealed that PGF_2a_ may play a crucial role in ovoviviparous teleost parturition, similar to other live bearing species, considering the function of this PG in mammalian myometrium contraction and in follicular apoptosis in teleosts (3, 60). The presence of immature dead larval fish confirmed the effect of PGF_2a_ on the unnatural induction of parturition in guppies.

In summary, the present study is the first to identify and characterize IT, AVT and ITRs in guppies. The results of the present study contributed to elucidation of direct effects of AVT and IT on *cox2* expression and subsequent induction of PG production in guppy mediated by CRE signaling, resulting in premature delivery. Our findings suggest that AVT functions as a more efficient factor that participates in parturition of live bearing teleost guppy by stimulating PG synthesis by upregulating *cox2* expression.

## Data Availability Statement

The data sets presented in this study can be found in online repositories. The names of the repository/repositories and accession number(s) can be found in the article/**Supplementary Material**.

## Ethics Statement

The animal study was reviewed and approved by Animal Research and Ethics Committees of Ocean University of China.

## Author Contributions

HSW, JFL, YL, and XQ designed the study. LKL performed the experiment. LKL, XJW, YJY, and JSL participated in the sample collection. LKL wrote the manuscript, and XQ provided feedback on the manuscript and edited the article All authors contributed to the article and approved the submitted version.

## Funding

This study was supported by grants from the National Key R&D Program of China (2018YFD0901204) and The National Natural Science Foundation of China (41976089 and 41676126).

## Conflict of Interest

The authors declare that the research was conducted in the absence of any commercial or financial relationships that could be construed as a potential conflict of interest.

## Supplementary Material

Supplementary Video 1 Representative video showing the first premature parturition of guppies injected with PGF_2a_ (1,000 ng/g); parturition occurred at 21 s.

## References

[1] Gimpl G Fahrenholz F. The oxytocin receptor system: structure, function, and regulation. Physiol Rev (2001) 81(2):629–83. 10.1152/physrev.2001.81.2.629 11274341

[2] Duffield A McKenzie C Carvalho B Ramachandran B Yin V El-Sayed YY. Effect of a High-Rate Versus a Low-Rate Oxytocin Infusion for Maintaining Uterine Contractility During Elective Cesarean Delivery: A Prospective Randomized Clinical Trial. Anesthesia analgesia (2017) 124(3):857–62. 10.1213/ane.0000000000001658 PMC5319709 28212181

[3] Kim SH Riaposova L Ahmed H Pohl O Chollet A Gotteland J-P. Oxytocin Receptor Antagonists, Atosiban and Nolasiban, Inhibit Prostaglandin F2α-induced Contractions and Inflammatory Responses in Human Myometrium. Sci Rep (2019) 9(1):5792. 10.1038/s41598-019-42181-2 30962532 PMC6453954

[4] Lavie A Shinar S Hiersch L Ashwal E Yogev Y Aviram A. Uterine electrical activity, oxytocin and labor: translating electrical into mechanical. Arch gynecology obstetrics (2018) 297(6):1405–13. 10.1016/j.ajog.2016.11.678 29453654

[5] Sakamoto T Nishiyama Y Ikeda A Takahashi H Hyodo S Kagawa N. Neurohypophysial Hormones Regulate Amphibious Behaviour in the Mudskipper Goby. PloS One (2015) 10(7):e0134605. 10.1371/journal.pone.0134605 PubMed PMID: 26230718 26230718 PMC4521927

[6] Ramsey ME Fry D Cummings ME. Isotocin increases female avoidance of males in a coercive mating system: Assessing the social salience hypothesis of oxytocin in a fish species. Hormones Behav (2019) 112:1–9. 10.1016/j.yhbeh.2019.03.001 30902535

[7] O’Connell LA Matthews BJ Hofmann HA. Isotocin regulates paternal care in a monogamous cichlid fish. Horm Behav (2012) 61(5):725–33. 10.1016/j.yhbeh.2012.03.009 22498693

[8] Almeida O Gozdowska M Kulczykowska E Oliveira RF. Brain levels of arginine–vasotocin and isotocin in dominant and subordinate males of a cichlid fish. Hormones Behav (2012) 61(2):212–7. 10.1016/j.yhbeh.2011.12.008 22206822

[9] Cunha-Saraiva F Balshine S Gozdowska M Kulczykowska E Wagner RH Schaedelin FC. Parental care and neuropeptide dynamics in a cichlid fish Neolamprologus caudopunctatus. Hormones Behav (2019) 116:104576. 10.1016/j.yhbeh.2019.104576 31442428

[10] Kleszczyńska A Sokołowska E Kulczykowska E. Variation in brain arginine vasotocin (AVT) and isotocin (IT) levels with reproductive stage and social status in males of three-spined stickleback (Gasterosteus aculeatus). Gen Comp Endocrinol (2012) 175(2):290–6. 10.1016/j.ygcen.2011.11.022 22137910

[11] Pickford GE Strecker EL. The spawning reflex response of the killifish, Fundulus heteroclitus: isotocin is relatively inactive in comparison with arginine vasotocin. Gen Comp Endocrinol (1977) 32(2):132–7. 10.1016/0016-6480(77)90143-5 892406

[12] Muchlisin ZA Arfandi G Adlim M Fadli N Sugianto S. Induced spawning of seurukan fish, Osteochilus vittatus (Pisces: Cyprinidae) using ovaprim, oxytocin and chicken pituitary gland extracts. Aquaculture Aquarium Conserv Legislation (2014) 7(5):412–8.

[13] Nainggolan M Napitupulu H Sipayung K Sukendi D. The Effect of Ovaprim Injections Combination With Oxytocin on Ovulation Stimulation and The Quality of Egg Hoven’s Carp (Leptobarbus Hoevenii Blkr). Int J Appl Environ Sci (2018) 13(7):621–32.

[14] Venkatesh B Tan CH Lam TJ. Prostaglandins and teleost neurohypophyseal hormones induce premature parturition in the guppy, Poecilia reticulata. Gen Comp Endocrinol (1992) 87(1):28–32. 10.1016/0016-6480(92)90146-b 1624095

[15] The role of arginine vasotocin in teleost fish osmoregulation. Symp Soc Exp Biol (2002) 2002(54):83–95. 10.2307/2332794 . 14992146

[16] Jurkevich A Grossmann R Balthazart J Viglietti-Panzica C. Gender-related changes in the avian vasotocin system during ontogeny. Microscopy Res Technique (2001) 55(1):27–36. 10.1002/jemt.1153 11596147

[17] Balment R Lu W Weybourne E Warne J. Arginine vasotocin a key hormone in fish physiology and behaviour: a review with insights from mammalian models. Gen Comp Endocrinol (2006) 147(1):9–16. 10.1016/j.ygcen.2005.12.022 16480986

[18] Foran CM Bass AH. Preoptic AVT immunoreactive neurons of a teleost fish with alternative reproductive tactics. Gen Comp Endocrinol (1998) 111(3):271–82. 10.1006/gcen.1998.7113 9707473

[19] Carneiro LA Oliveira RF Canario AV Grober M. The effect of arginine vasotocin on courtship behaviour in a blenniid fish with alternative reproductive tactics. Fish Physiol Biochem (2003) 28(1-4):241–3. 10.1023/b:fish.0000030542.31395.8a

[20] Ramallo MR Grober M Cánepa MM Morandini L Pandolfi M. Arginine-vasotocin expression and participation in reproduction and social behavior in males of the cichlid fish Cichlasoma dimerus. Gen Comp Endocrinol (2012) 179(2):221–31. 10.1016/j.ygcen.2012.08.015 22940647

[21] Acher R Chauvet J. The neurohypophysial endocrine regulatory cascade: precursors, mediators, receptors, and effectors. Front Neuroendocrinol (1995) 16(3):237–89. 10.1006/frne.1995.1009 7556852

[22] Song Z Albers HE. Cross-talk among oxytocin and arginine-vasopressin receptors: Relevance for basic and clinical studies of the brain and periphery. Front Neuroendocrinol (2018) 51:14–24. 10.1016/j.yfrne.2017.10.004 29054552 PMC5906207

[23] Hicks C Ramos L Reekie T Misagh G Narlawar R Kassiou M. Body temperature and cardiac changes induced by peripherally administered oxytocin, vasopressin and the non-peptide oxytocin receptor agonist WAY 267,464: a biotelemetry study in rats. Br J Pharmacol (2014) 171(11):2868–87. 10.1111/bph.12613 PMC4243861 24641248

[24] Ramos L Hicks C Caminer A Couto K Narlawar R Kassiou M. MDMA (‘Ecstasy’), oxytocin and vasopressin modulate social preference in rats: A role for handling and oxytocin receptors. Pharmacol Biochem Behav (2016) 150:115–23. 10.1016/j.pbb.2016.10.002 27725273

[25] Kawamata M Mitsui-Saito M Kimura T Takayanagi Y Yanagisawa T Nishimori K. Vasopressin-induced contraction of uterus is mediated solely by the oxytocin receptor in mice, but not in humans. Eur J Pharmacol (2003) 472(3):229–34. 10.1016/s0014-2999(03)01914-9 12871758

[26] Arrowsmith S Wray S. Oxytocin: its mechanism of action and receptor signalling in the myometrium. J Neuroendocrinol (2014) 26(6):356–69. 10.1111/jne.12154 24888645

[27] Kim SH Bennett PR Terzidou V. Advances in the role of oxytocin receptors in human parturition. Mol Cell Endocrinol (2017) 449:56–63. 10.1016/j.mce.2017.01.034 28119132

[28] Terzidou V Blanks AM Kim SH Thornton S Bennett PR. Labor and inflammation increase the expression of oxytocin receptor in human amnion. Biol Reprod (2011) 84(3):546–52. 10.1095/biolreprod.110.086785 PMC4480813 20926803

[29] Sztachelska M Ponikwicka-Tyszko D Sokolowska G Anisimowicz S Czerniecki J Lebiedzinska W. Oxytocin antagonism reverses the effects of high estrogen levels and oxytocin on decidualization and cyclooxygenase activity in endometrial tissues. Reprod BioMedicine Online (2019) 39(5):737–44. 10.1016/j.rbmo.2019.06.002 31548121

[30] Rebordão MR Galvão A Pinto-Bravo P Pinheiro J Gamboa S Silva E. Endometrial prostaglandin synthases, ovarian steroids, and oxytocin receptors in mares with oxytocin-induced luteal maintenance. Theriogenology (2017) 87:193–204. 10.1016/j.theriogenology.2016.08.028 27773348

[31] Gómez-Abellán V Sepulcre MP. The role of prostaglandins in the regulation of fish immunity. Mol Immunol (2016) 69:139–45. 10.1016/j.molimm.2015.09.022 26468035

[32] Sugimoto Y Inazumi T Tsuchiya S. Roles of prostaglandin receptors in female reproduction. J Biochem (2015) 157(2):73–80. 10.1093/jb/mvu081 25480981

[33] De Rensis F Saleri R Tummaruk P Techakumphu M Kirkwood R. Prostaglandin F2α and control of reproduction in female swine: a review. Theriogenology (2012) 77(1):1–11. 10.1016/j.theriogenology.2011.07.035 21958632

[34] Davis TL Bott RC Slough TL Bruemmer JE Niswender GD. Progesterone inhibits oxytocin-and prostaglandin F2alpha-stimulated increases in intracellular calcium concentrations in small and large ovine luteal cells. Biol Reprod (2010) 82(2):282–8. 10.1095/biolreprod.109.079970 PMC2809223 19812299

[35] Kuradomi RY Batlouni SR. PGF 2α and gonadal steroid plasma levels of successful and unsuccessful spawning Piaractus mesopotamicus (Teleostei, Characiformes) females. Aquaculture Int (2018) 26(4):1083–94. 10.1007/s10499-018-0269-8

[36] Stacey N. Effects of indomethacin and prostaglandins on the spawning behaviour of female goldfish. Prostaglandins (1976) 12(1):113–26. 10.1016/s0090-6980(76)80010-x 959573

[37] Sorensen PW Appelt C Stacey NE Goetz FW Brash AR. High levels of circulating prostaglandin F2α associated with ovulation stimulate female sexual receptivity and spawning behavior in the goldfish (Carassius auratus). Gen Comp Endocrinol (2018) 267:128–36. 10.1016/j.ygcen.2018.06.014 29940184

[38] Zhang Z Wen H Li Y Li Q Li W Zhou Y. TAC3 gene products regulate brain and digestive system gene expression in the spotted sea bass (Lateolabrax maculatus). Front Endocrinol (2019) 10:556:556. 10.3389/fendo.2019.00556 PMC6702303 31474940

[39] Bendtsen JD Nielsen H von Heijne G Brunak S. Improved prediction of signal peptides: SignalP 3.0. J Mol Biol (2004) 340(4):783–95. 10.1016/j.jmb.2004.05.028 15223320

[40] Southey BR Amare A Zimmerman TA Rodriguez-Zas SL Sweedler JV. NeuroPred: a tool to predict cleavage sites in neuropeptide precursors and provide the masses of the resulting peptides. Nucleic Acids Res (2006) 34(suppl_2):W267–72. 10.1093/nar/gkl161 PMC1538825 16845008

[41] Thompson JD Higgins DG Gibson TJ. CLUSTAL W: improving the sensitivity of progressive multiple sequence alignment through sequence weighting, position-specific gap penalties and weight matrix choice. Nucleic Acids Res (1994) 22(22):4673–80. 10.1007/978-1-4020-6754-9_3188 PMC308517 7984417

[42] Tamura K Stecher G Peterson D Filipski A Kumar S. MEGA6: molecular evolutionary genetics analysis version 6.0. Mol Biol Evol (2013) 30(12):2725–9. 10.2307/2413665 PMC3840312 24132122

[43] Qi X Zhou W Wang Q Guo L Lu D Lin H. Gonadotropin-Inhibitory Hormone, the Piscine Ortholog of LPXRFa, Participates in 17 β-Estradiol Feedback in Female Goldfish Reproduction. Endocrinology (2017) 158(4):860–73. 10.1210/en.2016-1550 28324026

[44] Kanda S Karigo T Oka Y. Steroid sensitive kiss2 neurones in the goldfish: evolutionary insights into the duplicate kisspeptin gene-expressing neurones. J Neuroendocrinol (2012) 24(6):897–906. 10.1111/j.1365-2826.2012.02296.x 22340198

[45] Qi X Zhou W Lu D Wang Q Zhang H Li S. Sexual dimorphism of steroidogenesis regulated by GnIH in the goldfish, Carassius auratus. Biol Reprod (2013) 88(4):81–7. 10.1095/biolreprod.112.105114 23467740

[46] Barberis C Mouillac B Durroux T. Structural bases of vasopressin/oxytocin receptor function. J Endocrinol (1998) 156(2):223. 10.1677/joe.0.1560223 9518866

[47] Huffman LS O’Connell LA Kenkel CD Kline RJ Khan IA Hofmann HA. Distribution of nonapeptide systems in the forebrain of an African cichlid fish, Astatotilapia burtoni. J Chem Neuroanat (2012) 44(2):86–97. 10.1016/j.jchemneu.2012.05.002 22668656

[48] Ribeiro D Nunes AR Gliksberg M Anbalagan S Levkowitz G Oliveira RF. Oxytocin receptor signalling modulates novelty recognition but not social preference in zebrafish. J Neuroendocrinol (2020) 32(4):e12834. 10.1111/jne.12834 31961994

[49] Pasquier J Cabau C Nguyen T Jouanno E Severac D Braasch I. Gene evolution and gene expression after whole genome duplication in fish: the PhyloFish database. BMC Genomics (2016) 17(1):1–10. 10.1186/s12864-016-2709-z 27189481 PMC4870732

[50] Martos-Sitcha JA Fuentes J Mancera JM Martínez-Rodríguez G. Variations in the expression of vasotocin and isotocin receptor genes in the gilthead sea bream Sparus aurata during different osmotic challenges. Gen Comp Endocrinol (2014) 197:5–17. 10.1016/j.ygcen.2013.11.026 24332959

[51] Lagman D Daza DO Widmark J Abalo XM Sundström G Larhammar D. The vertebrate ancestral repertoire of visual opsins, transducin alpha subunits and oxytocin/vasopressin receptors was established by duplication of their shared genomic region in the two rounds of early vertebrate genome duplications. BMC evolutionary Biol (2013) 13(1):1–21. 10.1186/1471-2148-13-238 PMC3826523 24180662

[52] Hasunuma I Toyoda F Okada R Yamamoto K Kadono Y Kikuyama S. Roles of arginine vasotocin receptors in the brain and pituitary of submammalian vertebrates. Int Rev Cell Mol Biol 304. Elsevier (2013) 304:191–225. 10.1016/B978-0-12-407696-9.00004-X 23809437

[53] Caldwell H Young H. Oxytocin and Vasopressin: Genetics and Behavioral Implications. In: Lajtha A., Lim R, editors. Handbook of Neurochemistry and Molecular Neurobiology. Springer, Boston, MA (2006). 10.1007/978-0-387-30381-9_25

[54] Albers HE. Species, sex and individual differences in the vasotocin/vasopressin system: relationship to neurochemical signaling in the social behavior neural network. Front Neuroendocrinol (2015) 36:49–71. 10.1016/j.yfrne.2014.07.001 25102443 PMC4317378

[55] Hawtin SR Wesley VJ Parslow RA Patel S Wheatley M. Critical role of a subdomain of the N-terminus of the V1a vasopressin receptor for binding agonists but not antagonists; functional rescue by the oxytocin receptor N-terminus. Biochemistry (2000) 39(44):13524–33. 10.1021/bi0013400 11063589

[56] Thibonnier M Coles P Thibonnier A Shoham M. The basic and clinical pharmacology of nonpeptide vasopressin receptor antagonists. Annu Rev Pharmacol Toxicol (2001) 41(1):175–202. 10.1146/annurev.pharmtox.41.1.175 11264455

[57] Duffy DM. Novel contraceptive targets to inhibit ovulation: the prostaglandin E2 pathway. Hum Reprod Update (2015) 21(5):652–70. 10.1093/humupd/dmv026 PMC4626744 26025453

[58] Crespo D Goetz FW Planas JV. Luteinizing hormone induces ovulation via tumor necrosis factor α-dependent increases in prostaglandin F 2α in a nonmammalian vertebrate. Sci Rep (2015) 5(1):1–12. 10.1038/srep14210 PMC4570979 26374476

[59] Martinović-Weigelt D Mehinto AC Ankley GT Berninger JP Collette TW Davis JM. Derivation and evaluation of putative adverse outcome pathways for the effects of cyclooxygenase inhibitors on reproductive processes in female fish. Toxicological Sci (2017) 156(2):344–61. 10.1093/toxsci/kfw257 PMC11017233 28201806

[60] Takahashi T Hagiwara A Ogiwara K. Prostaglandins in teleost ovulation: A review of the roles with a view to comparison with prostaglandins in mammalian ovulation. Mol Cell Endocrinol (2018) 461:236–47. 10.1016/j.mce.2017.09.019 28919301

[61] Joy K Singh V. Functional interactions between vasotocin and prostaglandins during final oocyte maturation and ovulation in the catfish Heteropneustes fossilis. Gen Comp Endocrinol (2013) 186:126–35. 10.1016/j.ygcen.2013.02.043 23510856

[62] Hausmann H Meyerhof W Zwiers H Lederis K Richter D. Teleost isotocin receptor: structure, functional expression, mRNA distribution and phylogeny. FEBS Lett (1995) 370(3):227–30. 10.1016/0014-5793(95)00832-T 7656982

[63] Lim S MacIntyre DA Lee YS Khanjani S Terzidou V Teoh T. Nuclear factor kappa B activation occurs in the amnion prior to labour onset and modulates the expression of numerous labour associated genes. PloS One (2012) 7(4):e34707. 10.1371/journal.pone.0034707 22485186 PMC3317641

[64] Joy KP Singh V. Functional interactions between vasotocin and prostaglandins during final oocyte maturation and ovulation in the catfish Heteropneustes fossilis. Gen Comp Endocrinol (2013) 186:126–35. 10.1016/j.ygcen.2013.02.043 23510856

